# Bone Phosphate

**Published:** 1871-01

**Authors:** 


### Editorial.

### BONE PHOSPHATE.

MANY fail to appreciate the wonderful properties of this salt. Its presence as the basis of bony tissue displays the wisdom of our Heavenly Father.

Phosphorus is a highly combustible element, and when 32 parts of it have combined with 40 of oxygen, it may be regarded as thoroughly burned, and the compound is called phosphoric acid. This acid unites with lime in several proportions, forming as many definite salts, called phosphates. Bone phosphate is a sub-salt, and is not the only subphosphate of lime. To guard against mistakes, we prefer the distinctive term used in our heading. This salt is decomposed with great difficulty. The acid and the base are not separated by a white heat, nor can other acids readily take lime from the phosphoric. Though nearly insoluble in water, it dissolves readily in hydrochloric acid, from which it may be readily displaced by an alkali, being precipitated without change. The gastric juice has no difficulty in dissolving it. It is very fortunate that a salt so permanent is so easily assimilated, in view of the fact that it is essential to the formation of the primary cells as well as the bony structures.

This salt is found in many articles of diet, as in the cereals, and in the muscular tissues of many animals, if not in all. It is probable that a deficiency of this salt in the food is a fruitful predisposing cause of dental decay. A lack of it in the teeth insures softness of the dentinal structure. This is the explanation of the fact that the teeth of the young are softer than those of the old.

A few practical suggestions may be of use here: Let there be a more intelligent and rational selection of dietary articles. The outer parts of the grains are richer in this salt than the inner. Let "cracked wheat" be a substitute for rice, and brown flour for "superfine," "family," "XXX," etc. Let beef, mutton, fowls, etc., take the place of pork and veal. Let milk, pure, fresh, and undiluted, become an article of every day use, especially with the young; and in extreme cases, as in pregnancy and lactation, don't hesitate to advise the free indulgence in pure bone phosphate with the food. And remember, too, that the organic portion of the tooth is quite as important as the inorganic; that if it is not properly developed, the lime salts, however abundant, will not be properly deposited in the dentinal tissue, so as to insure a hard, firm, strong, resisting tooth. Hence, bear in mind that good health during dentition is essential to the development of good healthy teeth. W.

"Mercury and carbonic acid are examples of bodies capable of passing from the vaporous to the crystalline state or condition by the management of pressure alone, so that crystal, soft-solid, waxy fluid, liquid and gaseous bodies may be exhibited of these substances, running up and down the scale of togetherness and apartness without cataclysm, flood or lines of demarkation of these differentiations, which are thus found to exist but in degrees commensurate to the pressure put on or taken off at pleasure of the investigator, or the spontaneity of natural production! *Pragmatic pathology* includes all the progresses and regresses in the constructions and destructions of the individual (separate) domains of crystalosophy, cellosophy and corpusculosophy, an exhaustive observation of whose processes will bring within the pale of consciousness all the possibilities of matter and mind.—*Report on Dental Pathology and Surgery.*

KEY TO THE ABOVE PERFORMANCE.

"That I may be clean, I feel bound to say to you, that not a word in that report is mine, but is taken from the fount of inspiration which wells up within me."—*Discussions on said Report.* See December number of DENTAL REGISTER.

And of such is (and is to be) the pathology (?) of the AMERICAN DENTAL ASSOCIATION! W.

